# Complete heart block during permanent His bundle pacing: Is it a novel complication?

**DOI:** 10.1016/j.hroo.2025.03.007

**Published:** 2025-03-17

**Authors:** Saad Ullah Malik, Pugazhendhi Vijayaraman

**Affiliations:** Division of Cardiac Electrophysiology, Geisinger Heart Institute, Wilkes Barre, Pennsylvania

We read the case report published by Reinoehl and colleagues^1^ with great interest. The authors describe a patient with persistent atrial fibrillation who underwent permanent pacemaker implantation with His bundle pacing (HBP). Immediately after HBP lead implantation, the patient developed complete heart block (CHB) and the planned atrioventricular (AV) node ablation was not performed. In this case, CHB persisted at 18 months. The authors presumed this to be the first such observed complication. While this is an interesting case report, we have the following observations regarding this publication:

This is not the first reported case of CHB after HBP lead implantation. In our previous publications, we had reported 4 cases of complete intra-Hisian block in a series of 450 consecutive patients undergoing permanent HBP.^2^^,^^3^

The overall risk of developing CHB associated with HBP is about 1.1%.^2^ In Reinoehl and colleagues’ series,^1^ 358 patients had no preexisting His-Purkinje conduction disease (normal HV intervals and narrow QRS), in whom acute His bundle injury in the form of CHB occurred in 4 (1.1%) patients. Conduction recovered within 5 to 15 minutes in 3 patients and at 24 hours in 1 patient. This phenomenon occurs when the His bundle is superficial (“naked” His bundle), and acute edema from direct lead fixation into the His bundle results in split His electrograms and HV block. Invariably, the His bundle capture threshold is <1 V in these patients. Since the original report, we have observed this phenomenon in a total of 12 patients in more than 1500 HBP lead implantations, in all of whom conduction had recovered within 24 hours. This suggests that this complication is a rare but reversible phenomenon.

In cases in which AV node ablation is planned but patient develops CHB during HBP lead implantation, ablation can still be pursued using the His-His interval as a surrogate for AV nodal conduction.^2^^,^^3^ Alternately, AV node ablation can be performed later when conduction recovers. However, conduction recovery did not occur in the case presented, leading to permanent CHB, which is the more unusual aspect of the case.
